# Mesostructured Silicas as Cation-Exchange Sorbents in Packed or Dispersive Solid Phase Extraction for the Determination of Tropane Alkaloids in Culinary Aromatics Herbs by HPLC-MS/MS

**DOI:** 10.3390/toxins14030218

**Published:** 2022-03-17

**Authors:** Lorena González-Gómez, Judith Gañán, Sonia Morante-Zarcero, Damián Pérez-Quintanilla, Isabel Sierra

**Affiliations:** Departamento de Tecnología Química y Ambiental, E.S.C.E.T, Universidad Rey Juan Carlos, C/Tulipán s/n, Móstoles, 28933 Madrid, Spain; lorena.gonzalez@urjc.es (L.G.-G.); judith.ganan@urjc.es (J.G.); sonia.morante@urjc.es (S.M.-Z.); damian.perez@urjc.es (D.P.-Q.)

**Keywords:** tropane alkaloids, solid-phase extraction, dispersive solid-phase extraction, strong cation exchange, mesostructured silicas, culinary aromatic herbs

## Abstract

In this work, Hexagonal Mesoporous Silica (HMS) and Santa Barbara Amorphous-15 (SBA-15) mesostructured silicas were synthesized and functionalized with sulfonic acid groups. The materials (HMS-SO_3_^−^ and SBA-15-SO_3_^−^) were evaluated as strong cation exchange sorbents for sample extract clean-up, by solid phase extraction (SPE) and dispersive solid phase extraction, to determine atropine (At) and scopolamine (Sc) in commercial culinary aromatic herbs. Under optimized conditions, 0.25 g of sample was subject to solid–liquid extraction with acidified water (pH 1.0), and good recovery percentages were achieved for At and Sc using 75 mg of HMS-SO_3_^−^ in SPE as the clean-up stage, prior to their determination by HPLC-MS/MS. The proposed method was validated in a thyme sample showing recoveries in the range of 70–92%, good linearity (R^2^ > 0.999), adequate precision (RSD ≤ 14%) and low limits (MDL 0.8–2.2 µg/kg and MQL 2.6–7.2 µg/kg for both analytes). Sixteen aromatic herbs samples (dried thyme, basil and coriander leaves) were analysed and At was found in fourteen samples over an interval of <5–42 μg/kg, whereas Sc was found in three of the sixteen samples studied (between <5–34 μg/kg). The amount of At and Sc found in some analysed samples confirms the importance of setting maximum levels of At and Sc in culinary aromatic herbs.

## 1. Introduction

Aromatic herbs have been used throughout history for a multitude of medicinal and culinary purposes [1,2]. Currently, aromatic herbs are widely used in modern cuisine and the industry as natural additives, because they improve the sensory properties of foods and help to extend their shelf life [3,4]. They are also sources of essential micronutrients, such as minerals and vitamins, and bioactive compounds [5]. For these reasons, their consumption is related to a decrease in chronic diseases, such as arthritis, asthma, cardiovascular diseases and cancer [4,5]. In addition, most of the aromatic herbs (for example, thyme, basil and coriander) are valuable as ingredients in infusions with a variety of uses, such as digestive, antispasmodic, antitussive, antidiarrheal, antiemetic, or anthelmintic [6]. Despite the beneficial properties of aromatic herbs, such as condiments and/or herbal infusions, the occurrence of some natural toxins in these foods, such as tropane alkaloids [7], pyrrolizidine alkaloids [8] or alkenylbenzenes [9], among others, should be controlled. Concerning alkaloids, contamination of aromatic herbs occurs mainly due to accidental co-harvesting of these herbs together with toxic plants or by horizontal natural transfer from alkaloid-containing living plants or plant material through the soil [8,9].

Tropane alkaloids (TAs) are secondary metabolites produced by different families of plants, mainly appearing in the Solanaceae family [10,11,12]. In particular, tropane-containing plants such as *Atropa, Datura* and *Hyoscyamus* are the main ones responsible for food contamination by these toxins. This is because they grow easily as weeds in crops of different plant foods. In addition, these alkaloids are found in all parts of the TAs-producing plants, seeds, leaves, roots and flowers, and cross contamination with the crops is usual, due to fast and mechanical harvesting [12]. For example, leaves of *D. stramonium* (jimsonweed or thorn apple) and *A. belladonna* (deadly nightshade) can be mistaken for leaves of other plants that are used to prepare salads or to make tea (for example mallow) due to physical resemblances [13].

The most outstanding compounds of the TAs big family, composed of more than 200 compounds, are (±)-hyoscyamine or atropine (At) and (−)-scopolamine (Sc). Intoxication with these compounds produces anticholinergic effects such as hallucinations, tachycardia, muscle spasms, and delirium, among others, causing a danger to human health [10]. In this regard, the European Food Safety Authority (EFSA) has appealed to the scientific community to collect more data on plant toxins, including TA levels in agricultural products susceptible to being contaminated by TA-producing plants [10,14]. This fact is important, taking into account the increasing interest in the consumption of plant products by the population, such as aromatic herbs and species, tea and herbal infusions. For example, in a recent EFSA report, significant amounts of TAs were reported in coriander seeds and thyme leaves samples [7]. In consequence, some plant-derived food products have been identified as relevant exposure sources of TAs for consumers, and a maximum level of 25 µg/kg as sum or At and Sc (50 µg/kg in anise seeds) has been established recently by the Commission Regulation (EU) 2021/1408 in dried herbal products used to prepare infusions [15].

Sensitive and reliable techniques are needed for the analysis of TAs in foods. In this sense, methods with a low quantitation limit (MQL) are recommended by the EFSA (5 µg/kg for agricultural products, ingredients, food supplements and infusions, 2 µg/kg for finished products and 1 µg/kg for cereal-based baby food products) [14]. Currently, some existing sample preparation protocols consist of simple solid–liquid extraction (SLE) with acidified aqueous solvents or mixtures of them with organic solvents [16,17,18,19,20,21,22]. Due to the complexity of food matrices, the sample extracts are sometimes purified and pre-concentrated by solid-phase extraction (SPE), in an arrangement of sorbent-packed columns. For this task, Strata X-C^®^ is currently one of the most widely used commercial cartridges [23,24,25,26]. The packed sorbent is a polymeric-type of material, modified with polar and sulfonic groups, that has been applied in sample extracts of flours, cereal-based products, teas and cereal-based baby foods.

Currently, trends in analytical chemistry highlight the evaluation of new materials in the sample preparation stage [12,27,28]. For example, the application of mesostructured silicas as new sorbents has been increased recently for food sample preparation, due to the advantages that they offer in terms of their textural characteristics, such as ordered structure with controlled pore size, large specific surface area and pore volume. In addition to the high chemical, mechanical and thermal stability, these silicas can be easily functionalized with organic ligands [29,30,31,32] or combined with other functional materials [33,34,35], which allows for its application for different purposes. In this sense, Santa Barbara Amorphous-15 (SBA-15) has been the main hexagonal mesostructured silica employed as sorbent for sample preparation. For example, in a recent work, sulfonic acid-functionalized SBA-15 has been satisfactorily used as strong cation exchange (SCX) phase in SPE (recoveries between 93–105%) for the analysis of TAs in flours and grains of pseudo-cereals, cereals and legumes. The mesostructured silica-based material showed better recoveries compared to a commercial SCX material, showing an alternative to conventional sorbents [36]. Other mesostructured silica with wormhole framework, such as Hexagonal Mesoporous Silica (HMS), has already been successfully proven for the determination of different contaminants, but to the best of our knowledge, HMS has never been evaluated for the analysis of natural toxins in food samples [37].

Therefore, the aim of this study was to evaluate for the first time the application of an HMS-type mesostructured silica functionalized with sulfonic acid groups (HMS-SO_3_^−^) as SCX sorbent in packed and dispersive SPE (dSPE). Results were compared with other mesostructured silica (SBA-15-SO_3_^−^), previously applied for SPE in the determination of TAs in flours and grains, in order to select the most adequate sorbent and protocol for the extraction of At and Sc in thyme, basil and coriander samples, prior to their determination by HPLC-MS/MS.

## 2. Results and Discussion

### 2.1. Characterization of Mesostructured Silicas

**Figure 1:**
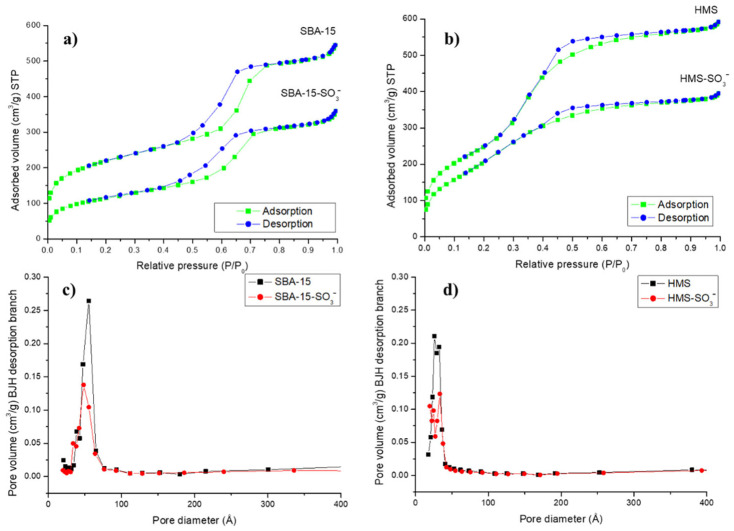
Nitrogen adsorption-desorption isotherms of bare and functionalized: (**a**) SBA-15 and (**b**) HMS materials. Pore size distribution of bare and functionalized: (**c**) SBA-15 and (**d**) HMS materials.

Nitrogen adsorption–desorption isotherms of the synthesized materials are shown in Figure 1. As can be seen, both the SBA-15 (Figure 1a) and the HMS (Figure 1b) showed isotherms type IV according to IUPAC classification with narrow hysteresis loop representative of mesoporous materials. SBA-15 showed an H1 hysteresis loop, indicative of uniform cylindrical pores, while HMS exhibited an H2 hysteresis loop, indicative of wormhole-like pores that cause some bottleneck effect. Pore size distribution of mesostructured silicas was calculated according to the Barret–Joyner–Halenda (BJH) method, obtaining narrow pore size distribution, which supplied evidence for uniform framework mesoporosity (Figure 1c,d).

Table 1 shows the physical parameters of nitrogen isotherms, such as the Brunauer–Emmett–Teller surface area (S_BET_), BJH average pore diameter and total pore volume for the mesostructured silicas. For both materials, high S_BET_ was observed, with typical average BJH pore diameter and pore volume values for surfactant-assembled mesostructured materials. HMS was the silica with the highest S_BET_ (911 m^2^/g), but with a small pore diameter (33 Å), while SBA-15 was the material with the lowest S_BET_ (780 m^2^/g) and the highest pore diameter (56 Å). As it can be seen in Table 1, a decrease in the S_BET_, average BJH pore diameter and pore volume were observed after functionalization in both cases that can be interpreted because sulfonic acid groups are attached not only on the silica surface but also inside the mesopores. The elemental analysis confirmed that silicas were successfully functionalized with sulfonic groups, obtaining a similar SO_3_^−^ density (L_0_) in both types of silica (around 1 mmol/g).

**Figure 2:**
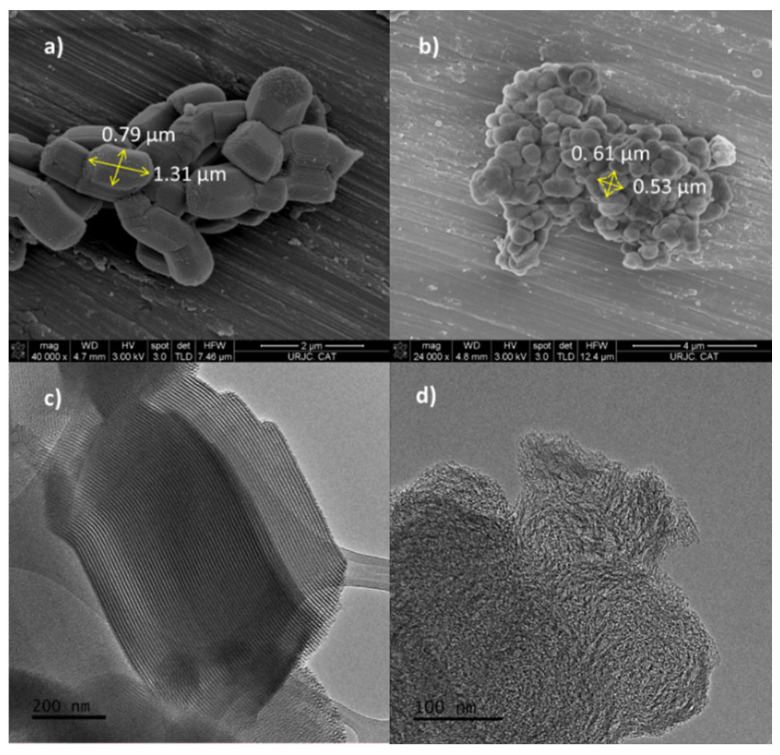
SEM images of: (**a**) SBA-15-SO_3_^−^ (40,000×) and (**b**) HMS-SO_3_^−^ (24,000×) materials. TEM images of: (**c**) SBA-15-SO_3_^−^ (200 nm) and (**d**) HMS-SO_3_^−^ (100 nm) materials.

SEM micrographs demonstrated the pseudo-spherical morphology of HMS particles with a medium particle size of 0.61 × 0.53 µm, while for SBA-15, the particle morphology was rope-like with a medium particle size of 0.79 × 1.31 µm that can be aggregated into a wheat-like macroscopic structure (Figure 2a,b). Figure S1 shows SEM images at a higher magnitude (80,000×). TEM pictures show a clear arrangement of ordered parallel pores with uniform sizes for SBA-15 (Figure 2c). In contrast, for HMS, TEM micrographs show irregularly aligned mesopores throughout the materials with relatively uniform pore sizes (wormhole-like pore arrangement) (Figure 2d). All materials kept their morphology, particle size and structure after functionalization.

### 2.2. Selection of the Best Conditions for Sample Treatment Procedure

SPE has been the most common technique used for the purification/pre-concentration of food sample extracts for the analysis of TAs [23,24,25,26], due to its simplicity and the variety of cartridges that can be achieved commercially. However, a disadvantage of SPE is that the cartridges must be uniformly packed with the sorbent, to form a packed bed to avoid poor extraction efficiency. This can be a difficult task when new sorbents materials are evaluated, and the cartridge is packed manually. Additionally, the particle size or shape of the sorbent may negatively influence mass diffusion and cause a drop in pressure of the extraction device. To overcome these problems, dSPE can be used since in this clean-up technique the sorbent material is directly added into the sample extract, thus improving the interaction area between the sorbent and analytes, which allows for using fewer amounts of sorbents and solvents.

With these considerations in mind, to evaluate the adsorption capacity of the synthesized materials, studies were carried out with standard solutions of At and Sc (0.01 and 0.001 µg/mL), comparing different amounts (100 and 150 mg) of SBA-15-SO_3_^−^ and HMS- SO_3_^−^ in two different clean-up techniques, dSPE and SPE. As is shown in Table 2, the results verify that satisfactory recoveries were achieved (>70%) with both materials and protocols, but, in general, recoveries were slightly higher with the SPE technique, especially with the SBA-15-SO_3_^−^ sorbent. This fact can be explained by the shape and size of the particles of the SBA-15-SO_3_^−^ material that clogs the pores of the nylon syringe filter, generating a high pressure in the filtering process in the dSPE protocol that made it difficult to completely elute the analytes. To confirm these results, the following experiments were carried out with a sample extract from thyme.

**Figure 3:**
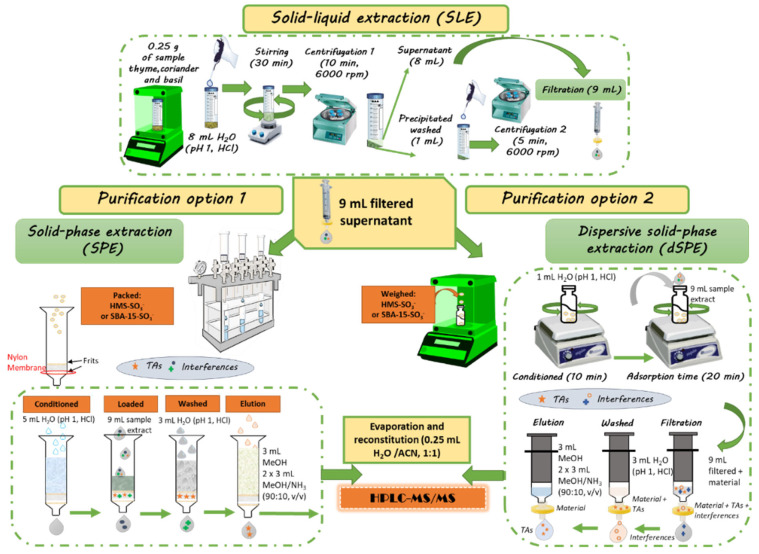
Schematic representation of the sample treatment procedure.

**Figure 4:**
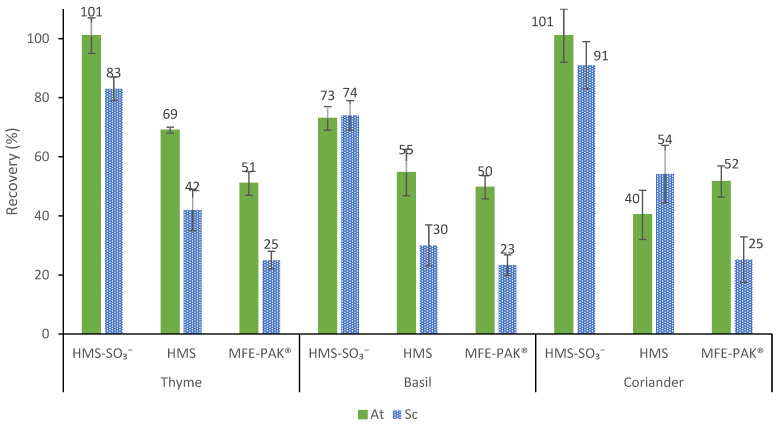
Recovery for atropine (At) and scopolamine (Sc) in thyme, basil and coriander spiked at 0.01 μg/g under the optimized conditions using 75 mg of HMS-SO_3_^−^, bare HMS silica and MFE-PAK^®^ commercial material.

First, 0.5 and 0.25 g of thyme sample were subject to SLE according to Section 4.6 (Figure 3). The acidified aqueous medium (H_2_O, 1.1% HCl, pH 1.0) was used as the extraction solvent, as it provided the best recovery for TAs in previous studies [36]. As TAs contain a ternary amine that is protonated at low pH, the relatively high solubility of the At and Sc in water enables a selective extraction with aqueous acid, excluding lipophilic compounds. Compared with other extraction media, acidified water is safe and cheaper. The resulting SLE extracts were purified by SPE or dSPE using 150 mg of both materials. To estimate recoveries, two thyme samples were doped prior to the extraction process, in order to achieve a concentration of 0.01 µg/g of At and Sc. The chromatographic areas were compared with that of thyme fortified extracts at the end of the sample treatment protocol. As it can be seen in Table 3, the best results were obtained with 0.25 g of sample, and the higher recoveries were observed with SPE clean-up with both materials (recoveries between 88 and 100%). Second, in order to miniaturize the clean-up protocols, the next studies were carried out with 0.25 g of thyme sample but with 100, 75 and 50 mg of sorbent. It can be seen in Table 3 that by reducing the amount of material from 150 to 100 mg, the recovery percentages decreased significantly with both sorbents in dSPE and with the SBA-15-SO_3_^−^ material in SPE. On the contrary, with the HMS-SO_3_^−^, sorbent no significant differences were observed in SPE. Good recoveries were also achieved with 75 mg of the HMS-SO_3_^−^ material in the SPE protocol (101% and 83% for At and Sc, respectively), but 50 mg resulted in not enough for a good recovery of the target analytes. In addition, as can be seen in Figure 4, good recoveries for basil and coriander samples (between 73 and 101%) were also achieved with 75 mg of the HMS-SO_3_^−^ material in the SPE protocol.

Finally, the performance of the bare silica was evaluated to demonstrate the contribution of the modifier in the retention process. Additionally, one last study was carried out to compare the HMS-SO_3_^−^ material with a commercial SCX sorbent (sulfonic acid groups bonded on amorphous silica). For this purpose, after SLE of 0.25 g of thyme, basil and coriander sample doped with 0.01 µg/g of TAs, extracts were purified by SPE with 75 mg of HMS or MFE-PAK^®^ materials. As can be seen in Figure 4, the non-functionalized silica afforded recoveries of 69 ± 1% and 42 ± 7% for At and Sc in thyme, respectively, and similar recoveries were obtained for basil (55 ± 8% and 30 ± 7% for At and Sc, respectively) and coriander (40 ± 8% and 54 ± 10% for At and Sc, respectively). With the commercial sorbent, even worse recoveries were obtained between 50–52% for At and between 23–25% for Sc in the three samples studied. At pH 1 in the bare HMS, ion–dipole interactions can occur, since at a pH lower than the point of zero charge (PZC, between 2–4), the surface charge of the material would be positive, and that allows ion–dipole interactions to be established with the polar groups of the TAs. Conversely, in the silica functionalized with sulfonic acid groups, HMS-SO_3_^−^ cation exchange interactions occur, which ensure better retention than in bare HMS. However, the differences observed in the recoveries between HMS-SO_3_^−^ and MFE-PAK^®^ can be attributed to the greater functionalization for HMS-SO_3_^−^ (1.015 mmol ligand/g) than for the MFE-PAK^®^ commercial sorbent (0.80 mmol ligand/g). Moreover, the good textural properties of the HMS silica (Table 1), such as high surface area, pore diameter and volume compared with the commercial amorphous silica, provide better accessibility to the functional groups and recovery percentages with this material. These results highlight the role of sulfonic groups in the ion-exchange interaction and reinforce the evidence found in previous studies on worse recoveries with commercial materials compared to mesostructured silica-based sorbents [36].

When comparing the results of both mesostructured sorbents, significant improvements were observed in the recovery values for At and Sc with the HMS-SO_3_^−^ silica and confirmed the best results obtained in SPE versus dSPE. These results may be attributed to the different textural properties of both materials. Despite having similar L_0_ (near 1 mmol/g), the smaller particle size with pseudo-spherical morphology of the HMS-SO_3_^−^ material provided better access to the 3D wormlike-framework-confined mesopores in adsorption processes in SPE, in comparison with the 2D hexagonally ordered cylindrical pores of the SBA-15-SO_3_^−^ material and, consequently, promote better retention of analytes with this clean-up procedure [38]. HMS materials were prepared for the first time by a neutral templating route, based on hydrogen bonding and self-assembly between neutral inorganic precursors and neutral primary amine micelles [39]. This templating route produces a mesostructured silica with thicker pore walls, which improve thermal and hydrothermal stability. In addition, the neutral and extensively cross-linked character of the HMS frameworks allows for, by simple solvent extraction, efficient and environmentally benign recovery of the cost-intensive template. Therefore, the HMS-SO_3_^−^ is a promising SPE sorbent for the extraction of TAs, and it is an alternative to classical commercial sorbents or to other mesostructured silicas such as SBA-15.

### 2.3. Method Validation

A sample of thyme, basil and coriander was selected to prepare matrix-matched calibration curves. Good linear regression for At and Sc was reached in all matrices (Table 4), obtaining R^2^ ≥ 0.999. Conversely, ME was calculated by comparison of slopes of solvent-based calibration and matrix-matched calibration, as indicated before. Signal suppression was observed for both compounds, mainly in the coriander sample (−61 and −71% of At and Sc, respectively). The selectivity was evaluated with a positive and blank sample to monitor interfering signals at the characteristic t_R_ for the TAs. First, it was verified that the t_R_ of both analytes in the sample extracts corresponded to that of the matrix-matched calibration standards, with a SD lower than ± 0.1 min. No signal was noted in the chromatogram of blank samples; thus, it can be concluded that the developed method was selective for the considered TAs (Figure S2). Ion ratios in unit mass resolution MS/MS were verified in positive samples and did not deviate more than 30% (relative abundance) from the value obtained in the corresponding spiked positive samples. Trueness and precision were evaluated in the thyme sample at three levels (Table S1). Good recovery percentages were obtained for At and Sc. At low and medium levels (5 and 25 µg/kg), recoveries of 90–92% were found for both analytes. At the high level (200 µg/kg), the recovery percentage decreased slightly to 87% for At and 70% for Sc, being acceptable values according to the SANTE/11813/2017 document [40]. Conversely, the precision showed RSD (%) ≤ 8% for At and ≤13% for Sc in intra-day at three levels and RSD (%) ≤ 8% for At and ≤14% for Sc in inter-day at three levels. Finally, MQL for At and Sc were according to limits recommended by EFSA in thyme and basil samples (Table 4), as they were ≤5 µg/kg [14]. On the contrary, in the coriander sample, MQL was 6.8 and 7.2 µg/kg for At and Sc, respectively.

Taking into account that plant-food products are complex samples, in analytical methods developed for TAs determination, the SLE extracts have usually been purified using commercial SPE cartridges, in order to reduce ME, improve accuracy and sensitivity. For example, Marin et al. [23] tested Strata^TM^-X-C and Oasis^®^ MCX cartridges to purify SLE-extracts of buckwheat, soy, millet and linseed samples, reaching the best recoveries with Strata^TM^-X-C (63–93%). However, even with the SPE purification step, an important ME was observed (matrix suppression between −60 to −80%) for At and Sc. In a recent study, González-Gómez et al. [36] demonstrated no ME after a clean-up SPE step with SBA-15-SO_3_^−^ of teff, corn and sorghum extracts, but significant ME was found in some other samples (for example in pea and amaranth with a matrix suppression between −35 to −69%). In this work, even with the purification SPE step using HMS-SO_3_^−^ as sorbent, a signal suppression was observed for both analytes, which was different for the three tested samples (lower in thyme and higher in coriander). This implies that to correctly quantify At and Sc in these samples, it was necessary to use the matrix-matched calibration curves for each type of aromatic herb to compensate for errors associated with matrix suppression. Conversely, Cirlini et al. [21] extracted TAs from herbal teas and supplements and they were analysed without a purification step (the extracts were only diluted with the extraction solvent). Although the recovery percentages were good (83–107% for At and Sc), the MQLs (25 µg/kg) were higher than the ones calculated in this study. This fact reflects that the incorporation of a purification/pre-concentration step allows for the establishment of lower MQLs for TAs in herbal matrices [10]. In addition, despite that methods can be applied without SPE processes, simplifying the analytical procedure in routine analysis, it is well known that by changing by SPE the matrix environment of the analyte of interest to a simpler matrix more suitable for subsequent analysis in the instrument, the chromatographic system lifetime is improved.

### 2.4. Analysis of Samples

**Figure 5:**
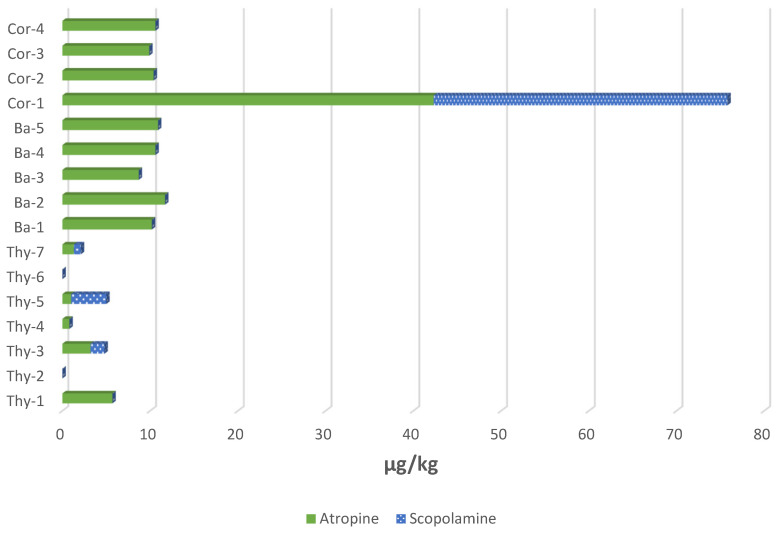
Content of TAs (At and Sc) found in sixteen commercial samples of thyme (Thy), basil (Ba) and coriander (Cor) analysed by the SLE-SPE-HPLC-MS/MS method proposed.

The optimized and validated SPE-HPLC-MS/MS method was applied, and in total, sixteen samples sold in Spain were analysed (seven of thyme, five of basil and four of coriander). For quantification, the areas obtained after the analysis of the extracts in the chromatograph were interpolated in matrix-matched calibration curves. Table S2 and Figure 5 show the content of At and Sc in the aromatic herbs analysed. To verify the complete extraction of At and Sc in contaminated samples, a double extraction with acidified water was carried out. The results obtained after this double extraction showed the absence of peaks for both analytes.

Of the sixteen samples, fourteen showed contamination with At, and the amounts quantified were between <5 and 42 µg/kg. Conversely, only three samples were positive in Sc; in two, this compound was detected but was below 5 µg/kg, and in the other, a concentration of 34 µg/kg was found. The highest TAs levels were found in the coriander and basil samples, and levels were clearly lower in thyme samples. In general, the At/Sc ratio observed was higher than 1, especially in coriander and basil samples (around 9–11) except for the Cor-1 sample with a ratio of 1.3. In the other samples, for example in Thy-5, the At/Sc ratio was lower than 1. All of these different ratios could be compatible with *Datura*, *Hyoscyamus* or *Atropa* sp. contamination, among others, as the amount of alkaloids is influenced not only by the plant species, but also by the part of the plant, the plant maturity and its geographical location [14]. For example, whereas thorn apple seeds from Italy have 14 times more At than Sc, in seeds from Poland, the content of At was 10–30% lower [13]. This fact shows that it is difficult to identify the possible source of contamination by plant-producing TAs and evidences the importance of monitoring commercially available foods, as occurrence data are essential to assess any potential risk of human toxicity. Now, maximum levels are only established for cereal-based foods for infants children (1 µg/kg for At and 1 µg/kg for Sc), some unprocessed and processed gluten-free cereals (between 5 and 15 µg/kg as sum of both TAs), dried products for herbal infusions (25 µg/kg as sum, 50 µg/kg in anise seed) and liquid herbal infusions (0.2 µg/kg as sum) [15]. In that respect, TAs level found in the sample Cor-1 (76 µg/kg sum of At and Sc) exceeded the maximum level allowed if used for infusions.

Few data are available regarding the presence of TAs in culinary aromatic herbs. In an EFSA document where human exposure to TAs was evaluated [7], large amounts of At were found in coriander seed samples (35.0 µg/kg) and small amounts of At and Sc were found in thyme (1.54 µg/kg). These values are in agreement with the levels of TAs found in our samples. For basil, no data have been found in this regard. Two recent Rapid Alert System in Food and Feed (RASFF) alert notifications [41], since 2020, have notified the presence of TAs in herbs and spices. One reported 66.7 ± 13.3 µg/kg (sum of At and Sc) in peppermint from Turkey and the other indicated a serious risk due to the presence of TAs in parsley samples from Hungary. The appearance of TAs in samples such as culinary aromatic herbs may be due to contamination by the leaves, and other parts, of the toxic plants of the Solanaceae family (*Datura stramonium*, *Datura innoxia*, *Atropa belladonna*, etc.) [10]. Aromatic plants as thyme, basil, and coriander typically yield two crops per year, one in summer and one in autumn, coinciding with the growth of TA-producing toxic plants. New harvesting practices, that is, the use of machinery, can lead to greater contamination of these toxic plants in aromatic herbs crops. In addition, visual inspection is difficult, especially in the finished product. This is because commercial products are sold in the form of crushed dry leaves; thus, if there is contamination by toxic plant parts, it is impossible to appreciate it. Given the toxicity of At and Sc and the high concentrations that can be found in this type of products, analysis of aromatic herbs is highly convenient to control the maximum levels set for both toxins in order to ensure a safe consumption of this type of products.

## 3. Conclusions

Two functionalized mesostructured silicas have been evaluated as cation exchange materials, in packed or dispersive SPE, for the determination of At and Sc in culinary aromatic herbs, prior to HPLC-MS/MS analysis. The best results were obtained with 75 mg of HMS-SO_3_^−^ of sorbent, using SPE to purify the extract, showing recoveries near to 100%. As far as we know, this is the first time that a mesostructured silica with worm-like pores (HMS type) has been successfully applied for this task. Sixteen commercial samples were analysed, and high amounts of At were found. It is worth highlighting the high TAs concentration observed in a coriander sample (76 μg/kg sum of At and Sc) that show the importance of controlling these toxins in aromatic herbs. Moreover, the application of the developed and validated method enables broad knowledge of the safety of the samples analysed, providing information of the main concerning TAs found in these scarcely studied culinary herbs.

## 4. Materials and Methods

### 4.1. Reagents and Materials

Dodecylamine 98% (DDA, MW = 185.35 g/mol; CAS 124-22-1), Tetraethylorthosilicate 98% (TEOS, MW = 208.33 g/mol CAS 78-10-4), poly(ethylene glycol)-block-poly (propylene glycol)-block-poly (ethylene glycol) (EO20PO70EO20, Pluronic^®^ 123, P123, MW = 5800 g/mol) were obtained from Sigma-Aldrich (St. Louis, MO, USA). (3-mercaptopropyl) triethoxysilane (MPTES) 94% was acquired from Alfa Aesar (Karlsruhe, Germany). Formic acid 99% Optima^™^ LC-MS grade was from Fisher Chemical (Madrid, Spain). Sodium chloride was from Panreac Química (Castellar del Vallès, Barcelona, Spain). Polyethylene frits (0.20 µm), nylon filter membranes (0.45 µm), empty syringes (3 mL) and nylon syringes filters (0.45 µm) was obtained from Scharlab (Barcelona, Spain). Ultra-pure water (resistance 18.2 MΩ cm) was obtained from a Millipore Milli-Q-System (Billerica, MA, USA). Acetonitrile (ACN) and methanol (MeOH) HPLC-MS grade, hydrochloric acid 37%, hydrogen peroxide 30%, ammonia solution 32%, and sodium hydroxide were acquired from Scharlab. Atropine sulfate ≥ 99% (At, CAS 51-55-8) and scopolamine hydrobromide ≥ 98% (Sc, CAS 6533-68-2) were purchased from Sigma-Aldrich (St. Louis, MO, USA). Stock standards solutions (1000 mg/L) were prepared by diluting 10 mg of At or Sc in MeOH and stored at −20 °C in darkness. Working standard solutions (0.005–10 mg/L) containing At and Sc were prepared by appropriate dilution of the stock solutions with ACN/H_2_O (50:50, *v*/*v*) and were stored at 2–8 °C.

### 4.2. Samples

Sixteen aromatic herb samples (dried leaves) were purchased from local supermarkets and herbalists in Madrid (Spain): seven samples of *Thymus vulgaris* (thyme, Thy), five samples of *Ocimum basilicum* (basil, Ba) and four samples of *Coriandrum sativum* (coriander, Cor). Sampling was performed according to the European Commission Regulation No. 401/2006 concerning sampling and analysis of mycotoxins in foodstuff [42]. All samples were separately milled with an A11 Basic analytical mill (IKA, Staufen, Germany) to obtain a fine powder, and then homogenized and stored at room temperature in the dark until their analysis. Each sample was analysed in triplicate.

### 4.3. Synthesis of Sulfonic Acid-Functionalized Mesostructured Silicas

Mesostructured silicas, SBA-15 and HMS, were first prepared according to previous works [43]. Then, both materials were functionalized according to the method described by González-Gómez et al. [36]. Briefly, 2.5 g of silica was suspended in 250 mL of 0.1 M HCl and 1.06 g of MPTES was added. The mixture was stirred at 180 rpm for 7 h, at room temperature, and transferred to an autoclave for 24 h at 100 °C. The resulting solid was filtered, washed with Milli-Q water and left at 50 °C during the night. The obtained materials (SBA-15-SH or HMS-SH) were mixed with 325 mL 2 M HCl, and next, 11.4 g of H_2_O_2_ (30%) was added for oxidation of thiols to sulfonic acid. After 5 min under stirring at room temperature, the mixture was heated in an autoclave for 6 h at 100 °C. The obtained materials, SBA-15-SO_3_^−^ or HMS-SO_3_^−^, were filtered and washed with Milli-Q water.

### 4.4. Characterization of Mesostructured Silicas Materials

X-ray diffraction (XRD) patterns of the silicas were obtained on a Philips diffractometer model PW3040/00 X’Pert MPD/MRD at 45 kV and 40 mA, using Cu-Kα radiation (λ = 1.55418 Å). Micrometrics ASAP 2020 analyser was used to obtain N_2_ gas adsorption-desorption isotherms. The surface specific area was calculated by Brunauer–Emmett–Teller (BET) method, and the pore size distribution was obtained using Barrett–Joyner–Halenda (BJH) model on the desorption branch. Scanning electron micrographs (SEM) were obtained on a Nova Nano SEM230 microscope with an energy dispersive spectrometry system. The samples were treated with a sputtering method with the following parameters: sputter time 100 s, sputter current 30 mA, and gold film thickness 3 nm using sputter coater BAL-TEC SCD 005. Conventional transmission electron microscopy (TEM) was carried out with a JEOL F200 ColdFEG microscope operating at 200 kV, with a resolution of 0.23 nm, using a copper sample holder. Flash 2000 Thermo Fisher Scientific Inc. analyser was used for the% S analysis.

### 4.5. Chromatographic Analysis of TAs

For the chromatographic analysis of TAs, a Varian 1200/1200L LC/MS-MS coupled to a Varian Prostar HPLC (Varian Ibérica, Spain) consisting of a ProStar 410 autosampler (equipped with a 100-μL loop), two ProStar 210/215 solvent deliver modules, a thermostatted column compartment and a 1200L TQ triple quadrupole mass spectrometry detector with an electrospray ionization (ESI) ion source (data acquisition system MS Workstation version 6.3) was used. A C18 Kromaphase 100 column (150 × 2.0 mm, 3.5 μm particle size) with a C18 Kromaphase guard column (10 × 4.0 mm I.D., 5 μm particle size) at 30 °C were obtained from Scharlab and were used for the chromatographic separation. The injection volume was 10 µL, and the flow rate was 0.25 mL/min. Solvent A (ACN) and solvent B (Milli-Q water), containing both 0.1% formic acid, were used as mobile phases in gradient elution mode. The gradient started at 10% A, and then it was increased linearly from 10% to 70% in 10 min and returned to 10% in 1 min (held for 4 min). The total run method was 15 min [36]. To prevent deterioration, the column was washed before use for 1 h with 100% ACN at a flow rate of 0.1 mL/min. Then, before analysing, it was conditioned for 20–30 min with the initial conditions of the gradient.

An electrospray ionization (ESI) ion source operating in positive mode was used for the mass-spectrometric analysis. N_2_ was used as both drying and nebulizer gas and argon was set as collision gas under the following conditions: N_2_ drying gas (350 °C, 22 psi), nebulizer gas pressure (58 psi), the capillary voltage (5000 V) and shield (600 V). Argon was set at 1.90 mTorr and detector voltage at 1480 V. Multiple reaction monitoring (MRM) mode was used for all analytes (mass peak width Q1 2.5; mass peak width Q3 2.5; scan width in MRM 0.70 s). Compounds were monitored at cone voltage of 70 V and Table S3 shows the optimal mass spectrum parameters (ion precursor, products ion and ions used for quantification).

### 4.6. Selection of the Best Conditions for Sample Treatment Procedure

First, in order to evaluate the cation-exchange extraction capacity of the sulfonic acid-functionalized silicas, both materials were evaluated as sorbents for SPE and dSPE using standard solutions of TAs. For this purpose, 150 and 100 mg of SBA-15-SO_3_^−^, or HMS-SO_3_^−^, materials were packed in SPE cartridges (3 mL), and extraction was carried out according to previous work [36]. Conversely, for dSPE, 150 and 100 mg of SBA-15-SO_3_^−^, or HMS-SO_3_^−^, silicas were weighed in a Falcon^®^ tube, and after the conditioning stage (1 mL of water acidified with 1.1% HCl, 10 min at 250 rpm), 9 mL of 0.01 or 0.001 µg/mL Tas standard solution were added, and the mixture was stirred for 20 min. After this time, the solvent was removed, and the sorbent was recovered using a 0.45 μm nylon syringe filter (25 mm of diameter, 0.45 μm of pore size). Next, the material was washed with 3 mL of water acidified with HCl (1.1%, pH 1.0), and TAs were eluted with 3 mL of MeOH, followed by 2 × 3 mL MeOH with ammonia solution (10%, pH 11.8). Eluates obtained in all protocols were evaporated to dryness and reconstituted with 250 μL of ACN/H_2_O (50:50, *v*/*v*) for subsequent chromatographic analysis.

Second, in order to optimize the sample treatment procedure, a thyme sample (0.25 or 0.5 ± 0.001 g) was weighed and extracted with 8 mL of water acidified with HCl (1.1%, pH 1.0) according to [36]. The supernatants obtained were filtered through a 0.45 µm nylon filter to be purified by SPE or dSPE, using 50, 75, 100 and 150 mg of SBA-15-SO_3_^−^, or HMS-SO_3_^−^, as sorbents, using the conditions indicated before (Figure 3).

### 4.7. Sample Treatment Procedure

First, 0.25 g (±0.001) of the sample was weighed in a Falcon^®^ tube and extracted with 8 mL of water acidified with HCl (1.1%, pH 1.0). The suspension was stirred for 30 min at room temperature. Then, it was centrifuged at 6000 rpm for 10 min, the supernatant was separated, washed with 1 mL of the extraction solution, and centrifuged for 5 min at 6000 rpm. The supernatants obtained were mixed (total amount: 9 mL) and filtered through a 0.45 µm nylon filter to be purified by SPE. For this purpose, 75 mg of HMS-SO_3_^−^ material was packed in 3 mL SPE cartridges. After conditioning with 5 mL of acidified water, the extract was loaded using a Scharlab ExtraVac^®^ (Barcelona, Spain) solid extraction vacuum manifold 12 port, at a flow rate of 1 mL/min. Next, cartridges were washed with 3 mL of acidified water and, finally, TAs were eluted at a flow rate of 1 mL/min with 3 mL of MeOH, followed by 2 × 3 mL of MeOH with ammonia solution (10%, pH 11.8). The eluate was evaporated to dryness and redissolved with 250 μL of ACN/H_2_O, (50:50, *v*/*v*) for subsequent chromatographic analysis.

### 4.8. Method Validation

The optimized SLE-SPE-HPLC-MS/MS method was validated in terms of linearity, matrix effect (ME), selectivity, trueness in terms of recoveries, intra- and inter- day precision, method detection (MDL) and method quantification (MQL) limits, following recommendation of the method validation guide for pesticide residues shown in the SANTE/11813/2017 document [40] and the IUPAC, ISO and AOAC International harmonised guidelines for single-laboratory validation of methods of analysis [44,45]. Thyme sample was used for full validation. Linearity was evaluated through matrix-matched calibration curves, which were prepared in three consecutive days by spiking the sample extracts with an adequate aliquot of a standard solution containing At and Sc to achieve the desired concentration level of the calibration curve. Six known concentration levels within the linear range were evaluated. At the same time, a blank sample or unspiked sample was used to correct the signal in case the sample was contaminated with TAs. The curves were constructed by plotting the peak area of each analyte against the concentration of the target analytes and were fitted by linear regression analysis. Linearity was evaluated through coefficients of determination (R^2^). To assess the ME, solvent-based standard calibration curves (between 0.005–10 μg/mL) were also prepared by using working standard solutions. The ME was calculated by comparing the slopes of both matrix-matched and solvent-based standard calibration curves as follows: ME = ((slope matrix-matched/slope solvent-based)—1 × 100). Positive and non-contaminated (blank) samples were used to evaluate the selectivity and to monitor interfering signals at the characteristic retention time (t_R_) for the TAs. Trueness was expressed in terms of recovery, spiking six samples (*n* = 6) with TAs at three concentration levels (low: 5 μg/kg, medium: 25 μg/kg and high: 200 μg/kg) at the beginning of the process, which were compared with a simulated sample doped at the end of the process. The low level was set according to the limit recommended by EFSA for aromatic herbs [14] and was near to the MQL of the method. The medium level was set according to the maximum levels of TAs established for dried products for herbal infusions by the Commission Regulation (EU) 2021/1408 [15]. Conversely, the precision was evaluated and expressed as relative standard deviation (% RSD). For this, intra-day precision was evaluated with six replicates in one day (*n* = 6) and for inter-day precision, three replicates in three different days (*n* = 9). Precision was also evaluated at three levels of concentration: high, medium and low. Lastly, MDL and MQL were determined as the 3-fold and 10-fold standard deviation of the peak area at the lowest concentration level. To quantify the TAs in the basil and coriander samples, matrix-matched calibration curves were prepared, and ME, MDL and MQL were also evaluated.

## Figures and Tables

**Table 1:** Characterization parameters for mesostructured silicas.

Silica	Channel Structure	Particle Morphology	L_0_ ^a^ (mmol SO_3_^−^/g)	S_BET_^b^ (m^2^/g)	BJH Pore Diameter ^c^ (Å)	Pore Volume ^d^ (cm^3^/g)
SBA-15	1D hexagonal parallel channels	Rope-like	-	780.0	56.0	0.80
SBA-15-SO_3_^−^	1D hexagonal parallel channels	Rope-like	1.026	567.8	49.0	0.65
HMS	3D wormhole-like	Pseudo-spherical	-	910.5	32.5	0.89
HMS-SO_3_^−^	3D wormhole-like	Pseudo-spherical	1.105	707.1	30.3	0.52

^a^ L_0_: functionalization degree. ^b^ S_BET_: specific surface area calculated by Brunauer–Emmett–Teller (BET) method. ^c^ BJH pore diameter: estimated by Barret–Joyner–Halenda (BJH) model applied in the desorption branch. ^d^ Pore volume: total pore volume measured at relative P/P_0_ = 0.97.

**Table 2:** Recovery percentages (% ± SD) for atropine (At) and scopolamine (Sc) in standard solutions using HMS and SBA-15 functionalized mesostructured silicas as dSPE or SPE sorbents.

Standard Solution (µg/mL)	Sorbent Amount (mg)	SBA-15-SO_3_^−^				HMS-SO_3_^−^			
		dSPE ^a^		SPE ^b^		dSPE ^a^		SPE ^b^	
		At ^c^	Sc ^d^	At ^c^	Sc ^d^	At ^c^	Sc ^d^	At ^c^	Sc ^d^
0.01	150	77 ± 7	85 ± 3	92 ± 1	118 ± 1	102 ± 11	101 ± 11	95 ± 6	99 ± 7
	100	72 ± 1	70 ± 2	95 ± 1	116 ± 2	79 ± 4	74 ± 8	80 ± 3	99 ± 8
0.001	100	85 ± 2	70 ± 2	97 ± 1	91 ± 2	83 ± 9	82 ± 8	93 ± 2	104 ± 5

^a^ dSPE: dispersive solid phase extraction; for experimental conditions see Figure 3. ^b^ SPE: solid phase extraction; for experimental conditions see section Figure 3. ^c^ At: atropine, recovery ± SD (%). ^d^ Sc: scopolamine, recovery ± SD (%).

**Table 3:** Recovery percentages (% ± SD) for atropine (At) and scopolamine (Sc) testing different amounts of thyme spiked with TAs at 0.01 µg/g under SLE * followed by dSPE or SPE, using different amounts of HMS or SBA-15-functionalized mesostructured silicas as sorbents.

Sample (g)	Sorbent (mg)	SBA-15-SO_3_^−^				HMS-SO_3_^−^			
		dSPE ^a^		SPE ^b^		dSPE ^a^		SPE ^b^	
		At ^c^	Sc ^d^	At ^c^	Sc ^d^	At ^c^	Sc ^d^	At ^c^	Sc ^d^
0.5	150	54 ± 2	37 ± 9	77 ± 3	61 ± 1	56 ± 2	49 ± 2	78 ± 2	74 ± 3
0.25	150	87 ± 8	73 ± 8	90 ± 3	88 ± 6	88 ± 8	79 ± 5	100 ± 3	98 ± 3
0.25	100	58 ± 0	43 ± 2	67 ± 3	48 ± 8	58 ± 10	47 ± 2	94 ± 3	82 ± 1
0.25	75	-	-	-	-	-	-	101 ± 6	83 ± 4
0.25	50	-	-	-	-	-	-	79 ± 7	64 ± 13

* SLE (solid–liquid extraction) conditions: 0.25 g thyme + 8 mL acidified water (pH 1.0, HCl), re-extraction with 1 mL of acidified water. ^a^ dSPE: dispersive solid phase extraction; for experimental conditions see Figure 3. ^b^ SPE: solid phase extraction; for experimental conditions see Figure 3. ^c^ At: atropine, recovery ± SD (%). ^d^ Sc: scopolamine, recovery ± SD (%)—not analysed.

**Table 4:** Linearity, matrix effect and limits for atropine and scopolamine analysis in thyme (Thy), basil (Ba) and coriander (Cor) samples with the optimized SLE-SPE-HPLC-MS/MS method.

		Atropine					Scopolamine				
Sample	Linearity (µg/mL)	Matrix Matched Calibration	R^2^	MDL ^a^ (µg/kg)	MQL ^b^ (µg/kg)	ME ^c^ (%)	Matrix Matched Calibration	R^2^	MDL ^a^ (µg/kg)	MQL ^b^ (µg/kg)	ME ^c^ (%)
Thy	0.005–0.2	5.8 × 10^8^ *x* + 9.9 × 10^5^	0.999	0.8	2.6	−36	2.0 × 10^8^ *x* + 3.4 × 10^5^	1.000	1.2	4.0	−43
Ba	0.005–0.2	4.5 × 10^8^ *x* + 9.5 × 10^5^	0.999	1.6	5.3	−50	1.4 × 10^8^ *x* + 2.1 × 10^5^	0.999	1.3	4.4	−59
Cor	0.01–0.2	3.6 × 10^8^ *x* − 1.7 × 10^6^	0.999	2.1	6.8	−61	1.0 × 10^8^ *x* − 6.5 × 10^5^	0.999	2.2	7.2	−71

^a^ MDL: method detection limit. ^b^ MQL: method quantification limit. ^c^ ME: matrix effect. To estimate ME, solvent-based calibration was prepared between 0.005–10 µg/mL (At: *y* = 9.1 × 10^8^ *x* + 5.8 × 10^7^; Sc: *y* = 3.5 × 10^8^ *x* + 5.5 × 10^7^).

## Supplementary Materials

The following supporting information can be downloaded at: https://www.mdpi.com/article/10.3390/toxins14030218/s1, Figure S1: SEM images of: (**a**) SBA-15-SO_3_^−^ (80,000×) and (**b**) HMS-SO_3_^−^ (80,000×) materials; Figure S2: Chromatograms of different product ions: (**a**) atropine and (**b**) scopolamine in standard solution (10 µg/L) and uncontaminated thyme sample (Thy-6), (**c**) atropine and (**d**) scopolamine in contaminated coriander sample (Cor-1); Table S1: Accuracy and precision of the method calculated for thyme sample; Table S2: Concentrations of atropine (At) and scopolamine (Sc) in commercial saFIGUREmples of thyme (Thy), basil (Ba) and coriander (Cor) with the proposed SLE-SPE-HPLC-MS/MS method; Table S3: Mass spectrum parameters for atropine (At) and scopolamine (Sc) using the developed HPLC-MS/MS method.
