# Supplementary material for: Mesostructured Silicas as Cation-Exchange Sorbents in Packed or Dispersive Solid Phase Extraction for the Determination of Tropane Alkaloids in Culinary Aromatics Herbs by HPLC-MS/MS
Source: Toxins (Basel). 2022 Mar 17;14(3):218. doi: 10.3390/toxins14030218 (PMC8948878; doi:10.3390/toxins14030218)
Supplement: Supplementary file 1 [file toxins-14-00218-s001.zip › toxins-1625463-supplementary.pdf]

Supplementary Materials: Mesostructured Silicas as Cation-Exchange Sorbents in Packed or Dispersive Solid Phase Extraction for the Determination of Tropane Alkaloids in Culinary Aromatics Herbs by HPLC-MS/MS

Lorena González-Gómez, Judith Gañán, Sonia Morante-Zarcero, Damián Pérez-Quintanilla and Isabel Sierra

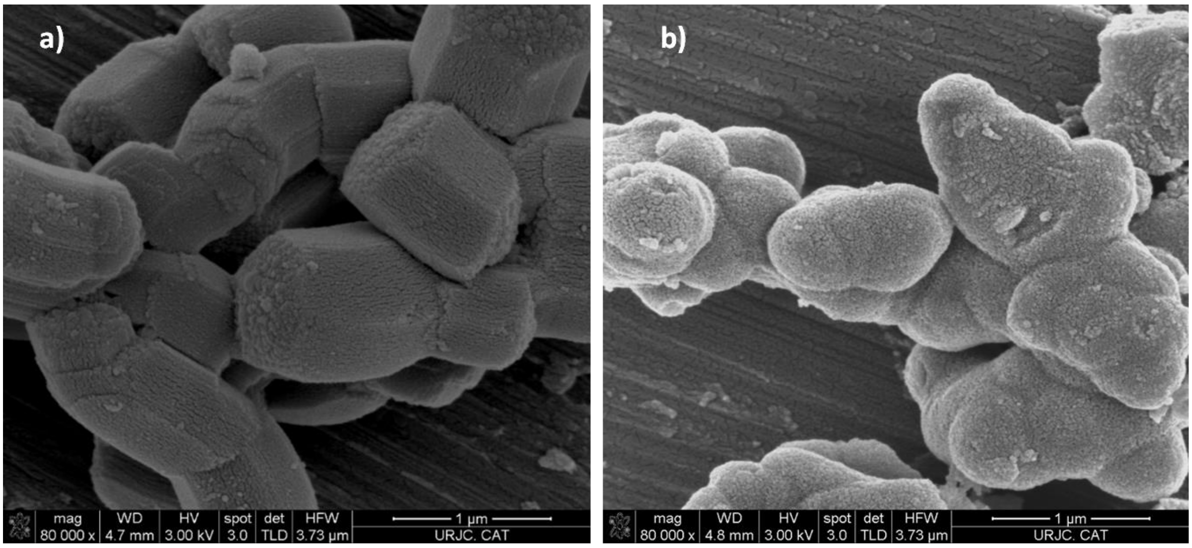

Figure S1. SEM images of: (**a**) SBA-15-SO_3^- (80,000×) and (**b**) HMS-SO_3^- (80,000×) materials.

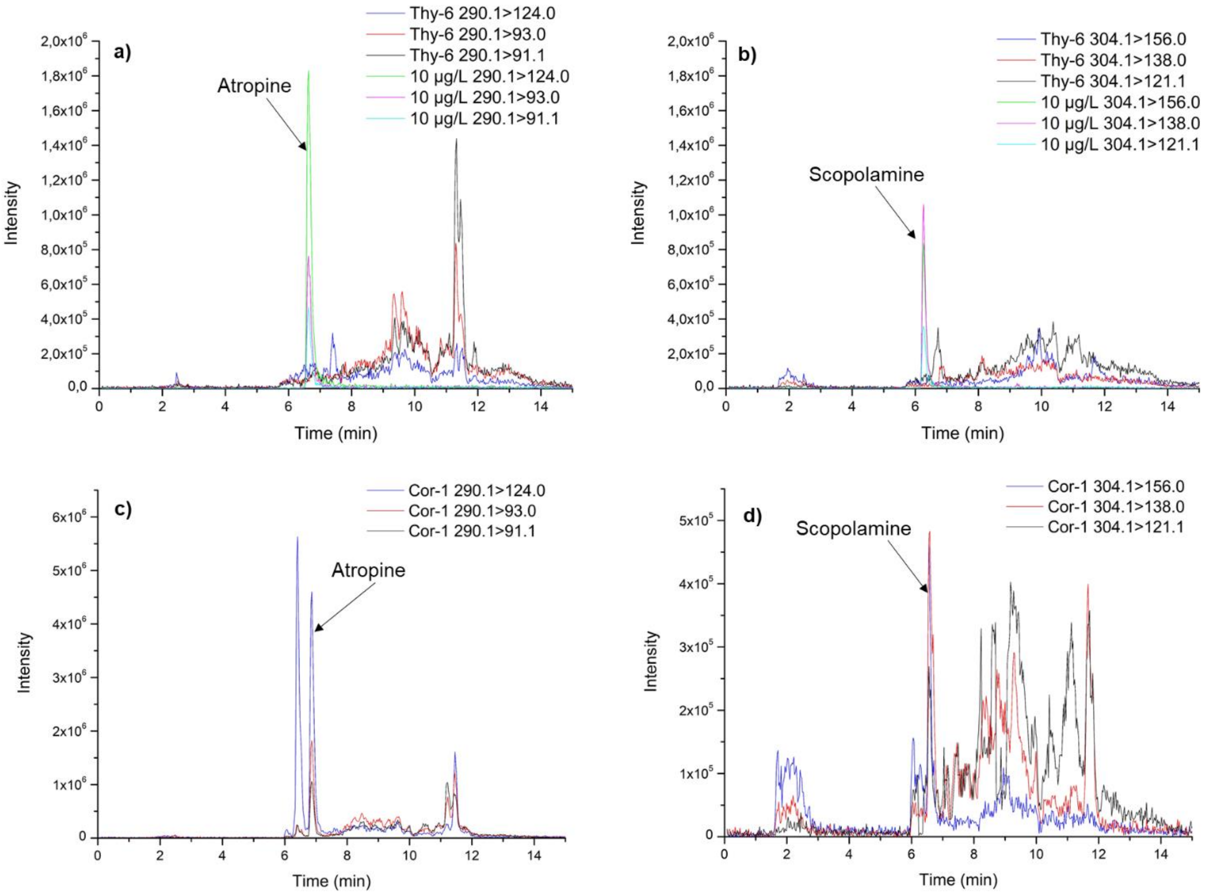

Figure S2. Chromatograms of different product ions: (**a**) atropine and (**b**) scopolamine in standard solution (10 µg/L) and uncontaminated thyme sample (Thy-6), (**c**) atropine and (**d**) scopolamine in contaminated coriander sample (Cor-1).

Table S1. Accuracy and precision of the method calculated for thyme sample.

	Level	Atropine	Scopolamine
	5 µg/kg	92 ± 6	92 ± 2
Accuracy (recovery % ± SD)[a]	25 µg/kg	92 ± 7	90 ± 7
	200 µg/kg	87 ± 2	70 ± 4
	5 µg/kg	8	13
Intra-Day Precision (%RSD)[b]	25 µg/kg	9	4
	200 µg/kg	3	2
	5 µg/kg	5	7
Inter-Day Precision (% RSD)[c]	25 µg/kg	8	11
	200 µg/kg	5	14

[a]Accuracy, expressed as recovery (%), was evaluated with six doped samples at the beginning of the process and compared with a simulated doped sample at the end. [b] Intra-Day Precision expressed as relative standard deviation (% RSD) for six replicates in one day (n = 6). [c] Inter-Day Precision eexpressed as relative standard deviation (% RSD) for three replicates in three different days (n = 9).

Table S2. Concentrations of atropine (At) and scopolamine (Sc) in commercial samples of thyme (Thy), basil (Ba) and coriander (Cor) with the proposed SLE-SPE-HPLC-MS/MS method.

Code	Origin	At (µg/kg)	Sc (µg/kg)
Thy-1	Poland	5.7 ± 0.2	N.D
Thy-2	Egypt/Poland	N.D	N.D
Thy-3	Spain	<5	<5
Thy-4	Spain	<5	N.D
Thy-5	Poland	<5	<5
Thy-6	Spain	N.D	N.D
Thy-7	Spain	<5	N.D
Ba-1	Poland	10.2 ± 0.4	N.D
Ba-2	Not shown	11.7 ± 0.4	N.D
Ba-3	Spain	9 ± 1	N.D
Ba-4	Egypt	11 ± 3	N.D
Ba-5	Egypt	11 ± 2	N.D
Cor-1	France/Germany	42 ± 5.0	34 ± 5
Cor-2	France/Germany	10.4 ± 0.9	N.D
Cor-3	Not shown	9.9 ± 0.2	N.D
Cor-4	Poland	10.6 ± 0.4	N.D

*N.D Not Detected.

Table S3. Mass spectrum parameters for atropine (At) and scopolamine (Sc) using the developed HPLC-MS/MS method.

Analyte	Ionization mode	Molecular weight (g/mol)	Precursor ions *m*/*z*	MS² Product ions *m*/*z*	Collision energy (V)	Dwell time (s)
At	ESI (+)	289.4	290	77	40	0.5
				91	34	
				93	28	
				124*	22	
Sc	ESI (+)	384.3	304	103	36	0.5
				121	20	
				138*	16	
				156	14	

* Ions used for quantification.
